# *Chromobacterium violaceum* and *Pseudomonas aeruginosa* PAO1: Models for Evaluating Anti-Quorum Sensing Activity of *Melaleuca alternifolia* Essential Oil and Its Main Component Terpinen-4-ol

**DOI:** 10.3390/molecules23102672

**Published:** 2018-10-17

**Authors:** Emira Noumi, Abderrahmen Merghni, Mousa M. Alreshidi, Ons Haddad, Gültekin Akmadar, Laura De Martino, Maha Mastouri, Ozgur Ceylan, Mejdi Snoussi, Abdulbasit Al-sieni, Vincenzo De Feo

**Affiliations:** 1 Laboratory of Bioressources: Integrative Biology & Recovery, High Institute of Biotechnology-University of Monastir, Monastir 5000, Tunisia; eb.noumi@uoh.edu.sa; 2 Department of Biology, College of Science, Hail, P.O. 2440, University of Ha’il, Hail 2440, Saudi Arabia; Mousa.alreshidi@uon.edu.au (M.M.A.); m.snoussi@uoh.edu.sa (M.S.); 3 Laboratory of Transmissible Diseases and Biologically Active Substances, Faculty of Pharmacy, University of Monastir, Monastir 5000, Tunisia; abderrahmen_merghni@yahoo.fr (A.M.); onshadad@gmail.com (O.H.); mastourimaha@yahoo.fr (M.M.); 4 University of Tunis El Manar, Faculty of Medicine of Tunis, Tunis 1007, Tunisia; 5 Ula Ali Kocman Vocational School, Mugla SitkiKocman University, Mugla 48147, Turkey; gultekinakdamar@windowslive.com (G.A.); ozgceylan@hotmail.com (O.C.); 6 Department of Pharmacy, University of Salerno, Via Giovanni Paolo II, 132, Fisciano, Salerno 84084, Italy; defeo@unisa.it; 7 Laboratory of Genetics, Biodiversity and Valorisation of Bioresources, High Institute of Biotechnology-University of Monastir, Monastir 5000, Tunisia; 8 Department of Biochemistry, Faculty of Science, King Abdul Aziz University, Jeddah 21589, Saudi Arabia; aalsieni@kau.edu.sa

**Keywords:** TTO, terpinen-4-ol, MRSA, biofilm, anti-quorum sensing, violacein, *Chromobacterium violaceum*, *Pseudomonas aeruginosa*

## Abstract

The problem of antibiotic resistance among pathogens encourages searching for novel active molecules. The aim of the research was to assay the anti-quorum sensing (anti-QS) and antibiofilm potential of *Melaleuca alternifolia* essential oil and its main constituent, terpinen-4-ol, to prevent the infections due to methicillin-resistant *Staphylococcus aureus* strains as an alternate to antibiotics. The tea tree oil (TTO) was evaluated for its potential in inhibiting QS-dependent phenomena such as violacein production in *Chromobacterium violaceum*, swarming motility of *Pseudomonas aeruginosa* PAO1, and biofilm formation in MRSA strains on glass. The results showed that terpinen-4-ol was able to inhibit MRSA strain biofilm formation on the glass strips by 73.70%. TTO inhibited the violacein production at a mean inhibitory concentration (MIC) value of 0.048 mg/mL by 69.3%. At 100 µg/mL TTO and terpinen-4-ol exhibited inhibition in swarming motility of PAO1 by 33.33% and 25%, respectively. TTO revealed anti-QS and anti-biofilm activities at very low concentrations, but it could be further investigated for new molecules useful for the treatment of MRSA infections.

## 1. Introduction

In the last years, the bacteria resistance to several antibiotics has increased in all parts of the world [1,2]. Thus, it is highly required to discover new different therapies to treat or decrease cases of bacterial infections. Due to the safety profile of some natural products, the interest in antimicrobials derived from plants has enhanced [3]. Plants have been used for centuries in popular medicine to cure infections and, nowadays, they continue to have an important role in the discovery of novel compounds [4]. Within secondary metabolites derived from plants, essential oils (EOs) contain bioactive components with chemical and structural differences and functions. For this motive, essential oils constitute a distinctive group of possible novel antimicrobial agents that have attracted particular interest [5].

Tea tree oil (TTO), an essential oil from *Melaleuca alternifolia* (Maiden & Betche) Cheel (Myrtaceae), has been known for its antimicrobial and anti-inflammatory effects. TTO consists of about 100 different components including terpinen-4-ol, which is one of its main antibacterial compounds [6]. Drugs and care products containing TTO are frequently used for the treatment of various infections or as antiseptics and disinfectants [6]. Preliminary clinical trials have demonstrated the efficacy of TTO in the elimination of methicillin-resistant *Staphylococcus aureus* (MRSA) [6]. Previous studies demonstrated the antimicrobial activity of TTO against *S. aureus* [7,8] and, therefore, this oil was widely adopted in the pharmaceutical industry and in the clinic [9]. *S. aureus* is able to form complex structures named biofilms. Bacteria in biofilms exhibit more resistance to antibiotics and host defense systems, which contribute to its pathogenesis and make the treatment of infections difficult [10].

The pathogen resistance and biofilm formation have been a goal of the efforts of researchers to find changes to antibiotic therapy [11]. An important approach is to target bacterial cell-to-cell communication, commonly known as quorum sensing (QS) [12] important in biofilm formation and in virulence factor production in different bacterial species [13]. It is well documented that uropathogens, such as *Pseudomonas aeruginosa*, possess a quorum sensing-controlled ability to form biofilms, which produces serious infections [14]. The biofilm formation is responsible for several QS-based factors, such as exopolysaccharide (EPS) production, swimming and swarming motility, and violacein production inhibition [15].

The aim of this research was to study, for the first time, the anti-quorum sensing activity of *M. alternifolia* essential oil using *Chromobacterium violaceum* and *Pseudomonas aeruginosa* PAO1 as bacterial models. Also, this study we aimed to report the chemical composition of the essential oil and the antibacterial activity as of the oil as of terpinen-4-ol against methicillin-resistant *S. aureus* strains. We indagate also the anti-MRSA biofilm potency of different concentrations of TTO and its main component terpinen-4-ol.

## 2. Results and Discussion

### 2.1. Essential Oil Composition

The composition of the essential oil of *M. alternifolia* is reported in Table 1. Fifteen components, listed according to their elution time on an HP5 capillary column, were identified, representing 93.8% of the oil. TTO was particularly rich in terpinen-4-ol (40.4%), γ-terpinene (19.5%), and α-terpinene (7.7%). Other relevant components were 1,8-cineole (5.2%), p-cymene (4.7%), α-terpineol (3.3%), and α-terpinolene (3.1%). Terpinenes and derivatives represent the great majority of the oil (74.0%). Our results agree with previous literature. More chemotypes of *M. alternifolia* have been reported, including terpinen-4-ol, terpinolene and four 1,8-cineole chemotypes [6,16]. Brophy and collaborators [17] used GC and GC/MS to analyze more than 800 TTO samples, with a great prevalence of terpinen-4-ol, γ-terpinene and α-terpinene. Also, commercial tea tree oil is very rich in terpinens [18]. This result agreed with a previous study which showed that terpinen-4-ol was the major component of a pure tea tree [19]. On the other hand, Russell and Southwell [20] found that the leaves of *M. alternifolia* contained a low quantity of terpinen-4-ol in the early stage of seedling growth and its concentration rose with plant development.

### 2.2. PCR Detection of the Sa442 and MecA Genes

Twenty-eight *Staphylococcus aureus* strains isolated from different pathological samples were identified using amplification of Sa 442 gene. Positive control (*S. aureus* ATCC 6538 and ATCC 43300) reacted appropriately. The Sa442 PCR assay was perfectly indicated for detecting *S. aureus*. Martineau and coworkers [21] identified a DNA segment, the Sa442 gene, characteristic of *S. aureus*. All the strains were confirmed to be MRSA harboring the mecA gene. In fact, the PCR assay for the mecA gene was perfectly indicated for detecting methicillin resistance in *S. aureus*. The mecA gene, the major determinant of methicillin resistance in staphylococci, [22,23]. This gene has been used for rapid identification of methicillin-resistant *S. aureus* (MRSA) also in bacterial subcultures.

### 2.3. Growth Inhibition Zones (mm) and MICs/MBCs Values (mg/mL) Determination

Our results showed that the essential oil of *M. alternifolia* and terpinen-4-ol were active against MRSA isolates with zones of inhibition ranging from 13 mm (strain Sa 21) to 37.5 (strain Sa4) for the TTO. Terpinen-4-ol is active against one MRSA clinical isolate (Sa24) and the two reference strains, similarly to the antibiotic Cefoxitin (5 µg/mL). Two isolates showed a resistance to terpinen-4-ol (Sa1 and Sa6) with a diameter of the zone of inhibition of 6 mm (Table 2).

Low concentrations of *M. alternifolia* essential oil and its main compound (terpinen-4-ol) inhibited the growth of all *S. aureus* strains tested independently of their origins. In fact, mean inhibitory concentration (MIC) values as low as 0.048–3.125 mg for the whole essential oil, and 0.048–1.52 mg/mL for the terpinen-4-ol are sufficient to inhibit the growth of all *S. aureus* strains tested. Whereas mean bactericidal concentrations (MBCs) about 25 to 50 mg/mL (whole volatile oil) and 6.25–50 mg/mL (terpinen-4-ol) are needed to completely inhibit the growth of the *S. aureus* strains tested.

In fact, TTO was reported for its antibacterial [24], antifungal [25] and antiviral [26] properties in vitro, suggesting that it could have an importance in the treatment of several infections. Furthermore, some clinical studies have revealed that TTO may be active in the treatment of oral candidiasis [27] and in the cure of infections by methicillin-resistant *Staphylococcus aureus* strains [28].

Although the in vitro antimicrobial activity and in vivo efficiency of TTO have been demonstrated, less is known about its mechanism of action against *S. aureus* [29].

### 2.4. Biofilm Formation on Abiotic Materials

In this study, polystyrene and glass were chosen because they are the most frequently used materials in medical devices. We have noticed that all MRSA strains independently of their colonization sites form a biofilm (0.1 < OD < 1 or OD_570_ > 1) on the tested materials with different degrees as function of the strain tested and the abiotic surface used (Table 3). In fact, four MRSA strains from different origins namely (Sa18, Sa30, Sa12 and Sa15) and the type strain *S. aureus* ATCC 6538 were high biofilm producers on polystyrene showing an OD > 1 (Table 3). The highest Biofilm producer strain on polystyrene material was the strain Sa12 (OD_570_ = 2.96 ± 0.11).

Twenty *S. aureus* strains out of 28 tested (71.43%) were high Biofilm producers on glass material with an optical density ranging from (1.01 ± 0.2) to (3.38 ± 0.12). Additionally, four strains (Sa18, Sa30, Sa12 and Sa15) were the highest biofilm-producers strains on both glass and polystyrene surfaces isolated from blood, superficial pus, and other origins. Vergara and coworkers [30] found that 22.7% of MRSA strains isolated from milk and meat were able to form biofilm on polystyrene and only one of these strains (1 out of 28, 4.5%) was able to produce biofilm on stainless steel (weak producer).

### 2.5. Anti-Adhesive Activity of M. alternifolia and Terpinen-4-ol on Polystyrene and Glass Surfaces

**Figure 1:**
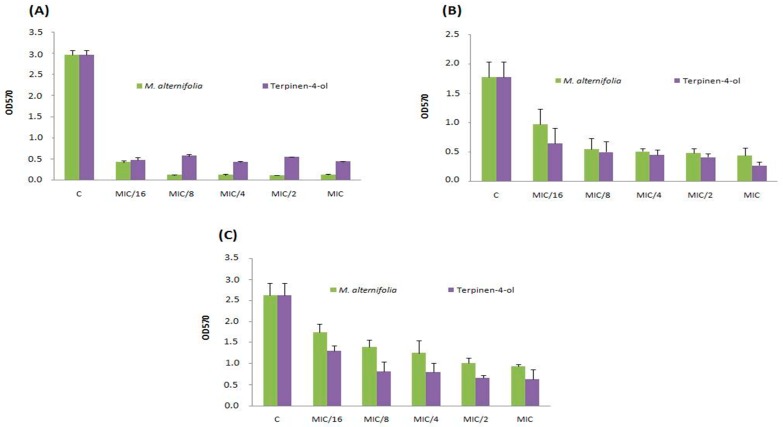
Anti-adhesive effects on polystyrene of TTO and terpinen-4-ol at different concentrations (MIC, MIC/2, MIC/4, MIC/8 and MIC/16) on three *S. aureus* strains including (**A**) strain Sa12; (**B**) strain Sa15 and (**C**) strain Sa18. C: control, OD: optical density.

To avoid biofilm formation is useful to prevent its colonization. Thus, we evaluated the ability of different concentrations of TTO and terpinen-4-ol to inhibit the adhesion. The study of anti-adhesive properties of TTO and terpinen-4-ol was carried out on three *S. aureus* strains namely Sa12, Sa15 and Sa18. These strains were selected according to their high biofilm production ability on both polystyrene and glass surfaces. TTO volatile and terpinen-4-ol showed anti-adhesive ability of MRSA strains on polystyrene at the lowest tested concentration (MIC/16 = 0.003 mg/mL) (Figure 1).

**Figure 2:**
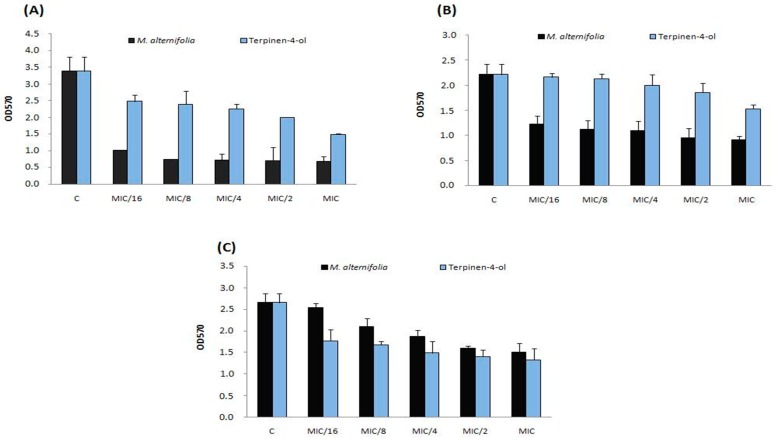
Anti-adhesive effects on glass of TTO and terpinen-4-ol at different concentrations (MIC, MIC/2, MIC/4, MIC/8 and MIC/16) on three *S. aureus* strains including (**A**) strain Sa12; (**B**) strain Sa15 and (**C**) strain Sa18. C: control, OD: optical density.

This effect was stronger against strain Sa12 compared to other strains. The anti-adhesive property has been observed on glass at the same concentration (MIC/16 = 0.003 mg/mL) and against the same isolate (Sa12, Figure 2). *M. alternifolia* essential oil was more active on sessile MRSA isolate Sa12 adherent into polystyrene and glass than its major compound (Figure 1A and Figure 2A).

### 2.6. Anti-Biofilm Activity of M. alternifolia Essential Oil and Terpinen-4-ol on Polystyrene and Glass

Resistance mechanisms of biofilms are multifactorial and depend on each organism. These mechanisms can be attributed to such factors as a reduction of the penetration of antibiotics through the biofilm matrix, the presence of slow-growing or non-growing cells in the biofilm, a heterogeneous bacterial population with the presence of phenotypic subpopulations with different levels of resistance, and the persistent presence of cells [31].

For determining the effect of TTO and its major compound on biofilm formation, we used the quantitative microtiter plate method. Three MRSA strains (Sa12, Sa15 and Sa18) were cultured in microtiter plates for 48 h in the presence of sub-inhibitory concentrations of the tested substances (MIC, 2 × MIC and 4 × MIC), and the formed biofilm was stained with crystal violet. Low concentrations (0.048, 0.096 and 0.192 mg/mL) of terpinen-4-ol showed a significant reduction of the biofilm formation, with a great percentage of inhibition of MRSA cells adhesion (Table 4).

**Figure 3:**
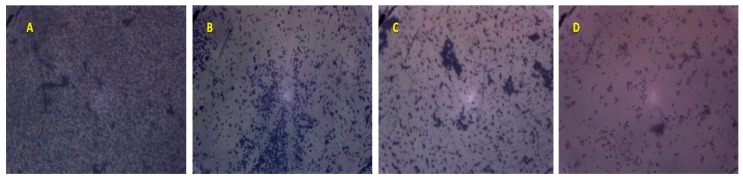
Effect of different concentrations of *M. alternifolia* essential oil on biofilm formation by Sa15 strain on glass at different concentrations. (**A**: Control; **B**: MIC; **C**: 2 × MIC and **D**: 4 × MIC), × 100.

The effect of TTO volatile oil on pre-formed biofilm was related to the concentration used and the strain tested. At 4 × MIC concentration, TTO essential oil was able to eradicate the pre-formed *S. aureus* (Sa15 strain) biofilm on both polystyrene and glass surfaces with a percentages ranging from (59.05 ± 2.83%) to (40.85 ± 1.61%), respectively (Figure 3).

High ability to eliminate the biofilm formed on both polystyrene and a glass material by using terpinen-4-ol was also recorded. In fact, the eradication percentage was up to (91.24 ± 5.81)% for Sa15 on polystyrene microplate and about (73.79 ± 9.47)% on glass material. The effects of natural antibacterial agents, such as berberine, carvacrol, oregano and thymol against staphylococcal biofilms have been frequently reported [32,33]. Other studies demonstrated that 1% of TTO is able to inhibit the biofilms formed by all isolates of *S. aureus* [34]. The biofilm destruction by TTO was due not only to bacterial killing but also to the destruction of the extracellular matrix and clearing of the biofilms from the surface [34].

### 2.7. Violacein Inhibition Assay in C. violaceum

**Figure 4:**
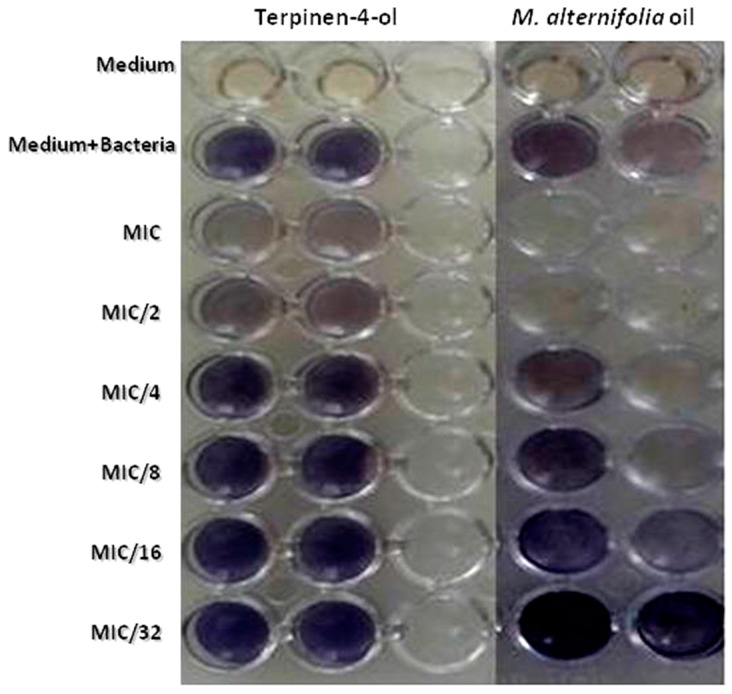
Effects of different MIC values of *M. alternifolia* and terpinen-4-ol on violacein inhibition (qualitative method with *C. violaceum* ATCC 12472).

The formation of *P. aeruginosa* biofilms is mostly regulated by Quorum Sensing [35]. To comprehend the mechanism of *P. aeruginosa* biofilm inhibition by TTO and terpinen-4-ol, we carried out the AHL-based in vitro QS competition assay with a suitable biosensor strain (*Chromobacterium violaceum*). In qualitative analysis, TTO showed an inhibition in HSL-mediated violacein production in *C. violaceum* ATCC 12,472 with a MIC of 69.3% and a MIC/2 of 58.98%. This inhibition was about 23.55% at a concentration of 0.3125 mg/mL (MIC/32) for the essential oil (Table 5). Terpinen-4-ol was able to inhibit violacein production with 17.87% until a concentration of about 2.5 mg/mL (MIC/4). (Table 5, Figure 4).

The anti-quorum sensing activities of some essential oils and their components have been investigated [36,37,38]. Khan and coworkers [39] reported that the sub-MIC of clove oil revealed 78.4% reduction in violacein production. Alvarez and coworkers [40] reported a >80% violacein inhibition by *M. alternifolia* oil at 0.50 µL/mL. Differences in properties of essential oils can often be attributed to variations in chemical composition. 

### 2.8. Swarming Inhibition Assay

As swarming migrations have an important role in biofilm formation mediated by Quorum Sensing, we examined the anti-QS potential of TTO and terpinen-4-ol on swarming motility in PAO1 test strain. The QS inhibiting activity of assayed substances was obtained from the levels of pyocyanin production in treated *P. aeruginosa*. Our results revealed that this essential oil inhibited the swarming of PAO1 at the three tested doses (50, 75 and 100 µg/mL) with pyocyanin concentration decrease between 16.67% and 33.33% (Table 6). However, a major inhibition in the migration of PAO1 was obtained at 100 µg/mL with a percentage of swarming inhibition about 33.33 % and 25 % for the TTO and terpinen-4-ol, respectively (Table 6). Khan and coworkers [39] reported that sub-MICs of clove oil revealed a decrease in swarming motility in *P. aeruginosa* PAO1 up to 78%.

## 3. Materials and Methods

### 3.1. Microorganisms

Twenty-eight clinical strains of methicillin-resistant *S. aureus* (MRSA) were selected to test the in vitro antibacterial activities of *M. alternifolia* essential oil and terpinen-4-ol. They were isolated from different pathological samples: superficial and deep pus (13 strains), blood culture (8 strains) and seven strains of various other specimens (tracheal aspiration, wound). These isolates were collected from Fattouma Bourguiba Hospital of Monastir (Tunisia). Two strains of *S. aureus* ATCC 6538 and ATCC 43300, were used as positive controls.

### 3.2. PCR Detection of the Sa 442 Gene

#### 3.2.1. Extraction of Bacterial DNA

There are different protocols for extracting DNA, but we have followed a simple protocol based on the method of lysis of the bacterial cells used in our laboratory. Thus, after inoculating the bacterial strains of *S. aureus* on nutrient agar and incubating for 18 to 24 h at 37 °C, some pure and well-isolated colonies were suspended in 1 mL of a solution of Tris-EDTA (TE), followed by a centrifugal washing of this suspension (13,200 rpm, 5 min at 4 °C). Subsequently, the supernatant was removed while the pellet was suspended in a volume of 200 μL TE, vortexed and then incubated for 10 min in a Marie bath at a temperature of 95 °C. After incubation, a final centrifugation step (13,200 rpm, 5 min at 4 °C) was made and the supernatant containing the bacterial DNA was moved into new Eppendorf tubes. The DNA was stored at 20 °C until use.

#### 3.2.2. PCR Detection of the Sa442 Gene

The PCR reaction was applied using the Sa442 gene. The primers used in this reaction were as follows: 5′-CGTAATGAGATTTCAGTAATAACAACA-3′ and 5′-AATCTTTGTCGGTACACG ATATTCTTCACG-3′ [41]. The size of the amplified fragments is of 218 bp. The total DNA was extracted using the lysis method. PCR was carried out in a total volume of 25 μL containing 50 ng of bacterial DNA, 5 μL of buffer (5×), 0.25 μL of dNTP (10 mM), 0.5 μL of MgCl_2_), 1 μL of each primer (25 μM), 1 U of GO Taq DNA polymerase (Promega, Fitchburg, WI, USA). The PCR mixture was placed in a thermocycler (Mastercycle). The PCR cycle conditions were as follows: initial denaturation at 94 °C for 5 min, followed by 35 cycles of denaturation at 94 °C for 90 s, hybridization at 57 °C for 30 s, elongation at 72 °C for 90 s and a final extension step at 72 °C for 10 min. PCR products (5 μL) were analyzed on 1.5% agarose gel with ethidium bromide (0.5 mg/mL) at 90 V for 1 h and visualized under ultraviolet light. The amplification products were photographed and their sizes were determined with a marker of molecular size of 100 bp (Invitrogen, Villebon-sur-Yvette, France).

#### 3.2.3. PCR Detection of the MecA Gene

The PCR detection of the mecA gene responsible for resistance to methicillin was carried out according to the technique described by Geha and coworkers [42]. The mecA primers are 5′-GTAGAAATGACTGAACGTCCGATAA-3′ (Forward) and 5′-CCAATTCCACATTGTTTCGGTCTAA-3′ (Reverse). The size of the amplified fragments is of 310 bp. The reaction mixture volume of the PCR was 25 μL, containing 200 μM of each dNTP (ATP, dTTP, dCTP, and dGTP); 0.5 μL of MgCl_2_, 1 U of GO Taq DNA polymerase (Promega, Fitchburg, WI, USA), 5 μL of Green Go Taq buffer (5×) and 1 μM of each primer. The cycle includes incubation at 94 °C for 5 min, 30 cycles of 1 min at 94 °C, 1 min at 55 °C and 2 min at 72 °C and a final extension of 72 °C for 10 min. The amplification was carried out in a PTC 100 thermocycler (Biorad, Marnes la Coquette, France). Then, the PCR product was analyzed by electrophoresis on a 2% agarose gel in 1× Tris-borate-EDTA buffer (TBE) and photographed by a Gel doc XR apparatus. The type strain *S. epidermidis* CECT 231 was used as negative control.

### 3.3. Chemical Characterization of the Essential Oil

*Melaleuca alternifolia* essential oil was purchased from Huile & Sens (Crestet, France). Terpinen-4-ol was purchased from Sigma (Sigma-Aldrich S.r.l. Milan, Italy). The essential oil was analyzed by gas chromatography–flame ionization detector (GC–FID) and gas chromatography–mass spectrometry (GC–MS). GC–FID analyses were performed using a Perkin Elmer Sigma-115 gas chromatograph with a data handling system and an FID. Analyses were carried out using an HP5 fused silica column (30 m × 0.25 mm i.d.; 0.25 µm film thickness). The operating conditions were as follows: injector and detector temperatures, 250 °C and 280 °C, respectively; oven temperature program, 5 min isothermal at 40 °C, then at 2 °C min^−1^ up to 250 °C and finally held isothermally for 20 min. Aliquots of 1 µL were injected manually at 250 °C and in the splitless mode. Analysis was also run by using a fused silica HP Innowax polyethylene glycol capillary column (50 m × 0.20 mm i.d.; 0.20 µm film thickness). In both cases, helium was used as the carrier gas (1 mL min^−1^). A diluted sample (1/100 *v*/*v*, in *n*-hexane) of 1 µL was injected manually at 250 °C and in the splitless mode. GC–MS analyses were carried out using a Hewlett-Packard 5890 A gas chromatograph connected on line to an HP mass selective detector (MSD 5970HP), equipped with an HP-1 fused-silica column (25 m × 0.25 mm i.d.; 0.33 µm film thickness); GC and GC–MS conditions: ionization voltage 70; electron multiplier energy 2000 V. Gas-chromatographic conditions were as reported above; transfer line was kept at 295 °C. Most constituents were identified by GC by comparison of their Kovats retention indices (Ri) (determined relative to the tR of *n*-alkanes (C_10_–C_35_)), with either those of the literature [43,44,45,46] and mass spectra on both columns with those of authentic compounds available in our laboratories by means NIST 02 and Wiley 275 libraries [47]. The component relative concentrations were obtained by peak area normalization. No response factors were calculated.

### 3.4. Antimicrobial Activities

#### 3.4.1. Disk Diffusion Assay

The antimicrobial activity testing was done according to the protocol described by Snoussi and coworkers [48]. Overnight cultures were used and the optical density was adjusted to 0.1 standard turbidity (OD_600 nm_). The inoculums were streaked onto MH agar plates using a sterile swab. Sterile filter discs (diameter 6 mm, Biolife, Milano, Italy) were impregnated with 10 mg of essential oil. Discs with the standard antibiotic, Cefoxitin (5 µg/mL), were taken as positive control. The plates were incubated at 37 °C for 18–24 h. The diameter of the zones of inhibition was taken as a measure of the antimicrobial activity.

#### 3.4.2. Microdilution Method for the Determination of the MIC and MBC

The minimal inhibition concentration (MIC) and the minimal bactericidal concentration (MBC) values were determined for all bacteria tested in this study using the microdilution assay [48]. The inoculums of the bacterial strains were prepared from an overnight culture and suspensions were adjusted to10^8^ cells/mL (DOλ = 600 nm). *M. alternifolia* essential oil and terpinen-4-ol were dissolved in 10% dimethylsulfoxide (DMSO 10%). The highest concentration was 50 mg/mL, and then serial twofold dilutions were prepared in concentrations ranging from 50 to 0.048 mg/mL in the 96-well plates and prepared by dispensing 100 µL aliquot from the stock solutions of each essential oil was added to 95 µL of the correspondent broth into the first well. Then, 100 µL from the serial dilutions were transferred into 10 consecutive wells. Finally, 5 µL of the inoculum of each microorganism was added to the wells. The last well, containing 195 µL of nutrient broth without essential oil and 5 µL of the inoculum on each strip was used as a negative control. The final volume in each well was 200 µL. The plates were incubated at 37 °C for 18–24 h.

### 3.5. Biofilm Production Assay by Staphylococcus Strains on Polystyrene, Glass and Stainless

*S. aureus* strains were grown in tryptic soy broth (TSB) and biofilm ability was studied using a semi-quantitative adherence assay on 96-well tissue culture plates (Nunc, Roskilde, Denmark) as described previously [49]. After incubation for 24 h at 37 °C, the absorbance at 570 nm (OD_570_) was recorded as a measure of total growth. An overnight culture grown in TSB (Biorad, Marnes la Coquette, France) at 37 °C was diluted to 1:100 in TSB with 2% (*w*/*v*) glucose to maximize *ica* operon induction [50]. A total of 200 µL of these cell suspensions was transferred in a U-bottomed 96-well microtiter plate. Each strain was tested in triplicate. Wells with sterile TSB alone was served as controls. *S. aureus* ATCC 25,923 and *S. aureus* ATCC 43,300 were used as a positive control. The plates were incubated aerobically for 24 h at 37 °C. Furthermore, the culture was removed, and plates were washed three times with 200 µL of phosphate-buffered saline (7 mM Na_2_HPO_4_, 3 mM NaH_2_PO_4_ and 130 mM NaCl at pH 7.4) to remove non-adherent cells and dried in an inverted position. Adherent biofilm was fixed with 95% ethanol and was stained with 100 µL of 1% (wt vol^−1^) crystal violet (Merck, Lyon, France) for 5 min. Then, unbound crystal violet was removed and the wells were washed three times with sterile distilled water. The water was then absorbed and the microtiter plate was air dried for 30 min. The optical density (OD) of each well was measured at 570 nm using an automated Multiskan reader (Gio De Vita, Rome, Italy). Biofilm formation was categorized as highly positive (OD_570_ ≥ 1), low-grade positive (0.1 ≤ OD_570_ < 1), or negative (OD_570_ < 0.1).

For biofilm formation on stainless and glass, the strips (1.5 cm^2^) were disinfected by dipping in 70% alcohol for 30 min and washed with sterile distilled water. They were then ultrasonicated for 20 min to remove any contaminants and artefacts from the surfaces, washed again in sterile distilled water, dried and used for the biofilm assay. Successively, strips were placed into 12-well tissue culture plate and 100 µL of bacterial suspensions were placed into each strip. The cells were allowed to adhere for 24 h at 37 °C. Non-adherent cells were removed from the strips by being gently washed with 5 mL PBS. Strips to which no cells were added served as negative controls. Biofilm quantification was made with crystal violet 1% staining and then dissolved into acetic acid (33%). One hundred twenty-five μL of each well were transferred on 96-well microtiter plate and the OD at 570 nm was measured [51].

### 3.6. Determination of Anti-Biofilm Anti-Adhesive Activities on Polystyrene and Glass

MIC, 2 × MIC and 4 × MIC of *M. alternifolia* essential oil and terpinen-4-ol were tested for their anti-*Staphylococcus* biofilm formation. Only three strains were selected for this test. A crystal violet assay was employed to test the effects on biofilm formation. One hundred µl of fresh bacterial suspension was added to each well. Growth control (cells + broth), media control (only broth) and blank control (broth + extract) were included. After incubation at 37 °C for 24 h, the biofilm biomass was evaluated using the crystal violet staining assay as described above and the OD at 570 nm was measured [51].

### 3.7. Violacein Inhibition Assay

*C. violaceum* ATCC 12,472 was used in the qualitative screening of violacein inhibition. Ten microliters of an overnight culture of *C. violaceum* ATCC 12,472 (OD adjusted to 0.4 at 600 nm) was added into wells of sterile microtiter plates containing 1 mL of LB broth and incubated in the presence and absence of various concentrations of TTO and terpinen-4-ol (MIC = 10 mg/mL until MIC/32 = 0.3125 mg/mL) at 30 °C for 18 h and observed for inhibition of violacein production [52].

### 3.8. Swarming Assay

In swarming assay, overnight cultures of *P. aeruginosa* PAO1 strain were point inoculated at the center of swarming plates consisting of 1% peptone, 0.5% NaCl, 0.5% agar and 0.5% of filter-sterilized d-glucose with various concentrations of *M. alternifolia* essential oil and terpinen-4-ol (50, 75 and 100 µg/mL) and the plate without the extract was maintained as control. Plates were incubated at an appropriate temperature in an upright position for 18 h [52]. The swarming migration was recorded by following swarm fronts of the bacterial cells.

## 4. Statistical Analysis

All biological assays (disc diffusion, biofilm formation, violacein inhibition and anti-swarming potency) were expressed as Means ± Standard Deviation (SD). Each analysis was performed using the SPSS 16.0 statistics package for Windows.

## 5. Conclusions

This study provided strong evidence that tea tree oil (TTO) could be used as an alternative treatment for bacterial infections, in particular those associated with biofilm formation. This study specifically concluded that the low concentration of TTO was able to eliminate biofilm formation and subsequently cells communication (quorum sensing). However, it is highly recommended to further search for new effective compounds for the treatment of bacterial infection.

This section is not mandatory, but can be added to the manuscript if the discussion is unusually long or complex.

## Figures and Tables

**Table 1:** Chemical composition of the essential oil of *M. alternifolia* as determined by using the GC-MS technique.

N.		Ki ^a^	Ki ^b^	% ^c^	Identification ^d^
**1**	α-Thujene	916	930	0.9	1,2
**2**	α-Pinene	921	1032	2.7	1,2,3
**3**	β-Pinene	980	979	0.7	1,2,3
**4**	Myrcene	985	990	0.8	1,2
**5**	α-Terpinene	1010	1189	7.7	1,2,3
**6**	*p*-Cymene	1018	1269	4.7	1,2,3
**7**	1,8-Cineole	1024	1213	5.2	1,2,3
**8**	γ-Terpinene	1054	1256	19.5	1,2,3
**9**	α-Terpinolene	1083	1265	3.1	1,2
**10**	α-Terpineol	1180	1188	3.3	1,2,3
**11**	Terpinen-4-ol	1173	1611	40.4	1,2,3
**12**	Isoledene	1382	1367	1.2	1,2
**13**	Aromadendrene	1442	1628	0.5	1,2
**14**	*allo*-Aromadendrene	1458	1661	1.5	1,2,3
**15**	δ-Cadinene	1523	1773	1.5	1,2
	Total			93.8	

^a^ Kovats retention index determined relatively to the t_R_ of a series of *n*-alkanes (C_10_–C_35_) on HP-5 MS column. ^b^ Kovats retention index determined relatively to the t_R_ of a series of *n*-alkanes (C_10_–C_35_) on HP Innowax. ^c^ t = trace (<0.1%). ^d^ 1 = Kovats retention index, 2 = mass spectrum, 3 = co-injection with authentic compound.

**Table 2:** Anti-*Staphylococcus* activity of tea tree oil TTO essential oil and terpinen-4-ol (mean inhibitory concentration (MIC) and minimum bactericidal concentration (MBC) values in mg/mL).

Strains	*M. alternifolia*			Terpinen-4-ol			Cefoxitin (5 µg/mL)
	DZI * (mm ± SD)	MIC	MBC	DZI (mm ± SD)	MIC	MBC	
ATCC 6538	26 ± 0	0.048	50	23.5 ± 0.7	0.048	50	26
ATCC 43300	23.33 ± 1.89	0.048	50	22 ± 2	0.048	25	26
Sa1	19.33 ± 0.58	0.78	25	6 ± 0	0.78	6.25	21
Sa3	19.67 ± 0.58	3.125	>50	25 ± 0	0.048	25	21
Sa4	37.5 ± 0.7	3.125	>50	20.67 ± 0.58	0.048	25	14
Sa16	18.67 ± 0.58	0.048	>50	20 ± 0	0.048	25	21
Sa17	20.5 ± 1	0.048	>50	24 ± 1	0.048	25	21
Sa18	27 ± 1	0.048	>50	25 ± 1	0.048	25	21
Sa21	13 ± 1	0.048	50	29.67 ± 0.58	0.048	6.25	14
Sa26	19.33 ± 0.58	0.048	>50	23.33 ± 0.58	0.048	50	21
Sa5	20 ± 1	0.048	>50	29.67 ± 0.58	0.048	25	21
Sa6	21 ± 1	0.048	>50	6 ± 0	0.048	25	17
Sa7	19.67 ± 0.58	0.048	>50	24.33 ± 2.08	0.048	25	21
Sa13	28.5 ± 0.7	0.048	>50	28.33 ± 0.58	0.048	25	18
Sa24	25.5 ± 0.7	0.048	>50	30.33 ± 0.58	0.048	25	21
Sa25	17.67 ± 0.58	0.048	>50	20 ± 1	0.048	25	19
Sa28	19.33 ± 0.58	0.048	>50	21 ± 1	0.097	25	21
Sa29	20 ± 0	0.048	50	22 ± 0	0.048	25	21
Sa30	19.33 ± 0.58	0.048	>50	20 ± 0	0.048	25	14
Sa31	13.5 ± 0.7	0.048	50	26 ± 1	0.048	50	8
Sa32	16.5 ± 0.7	0.048	>50	18.5 ± 0.7	0.048	50	20
Sa9	27 ± 1	0.048	>50	29.33 ± 0.58	0.048	25	14
Sa27	19.67 ± 0.58	0.048	>50	21.33 ± 0.58	0.048	50	16
Sa2	18 ± 0	0.78	>50	29.67 ± 0.58	0.048	25	21
Sa8	26.67 ± 0.58	0.048	>50	29.33 ± 0.58	0.048	25	20
Sa10	27 ± 1	0.048	>50	30 ± 0	0.048	25	6
Sa12	18.5 ± 0.7	0.048	>50	26 ± 0	0.048	25	21
Sa15	28 ± 0	0.048	>50	25 ± 0	0.048	25	6
Sa19	25 ± 0	0.048	50	20.67 ± 0.58	1.52	25	19
Sa23	26.5 ± 0.7	0.048	50	21 ± 0	0.048	25	21

*: Diameter of inhibition zones of compound including diameter of well 6 mm. The data are expressed as mean ± SD (*n* = 3). MIC: Minimal inhibitory concentration (mg/mL); MBC: Minimal bactericidal concentration (mg/mL).

**Table 3:** Biofilm formation on abiotic surfaces (polystyrene and glass) by *S. aureus* strains estimated by the Cristal Violet technique.

Strains	Origin	Biofilm on Polystyrene		Biofilm on Glass	
		OD_570_ ± SD	Biofilm Potency	OD_570_ ± SD	Biofilm Potency
ATCC 6538	Type strain	2.90 ± 0.05	High producer	2.23 ± 0.5	High producer
ATCC 43300	Type strain	0.71 ± 0.15	Low producer	2.98 ± 0.4	High producer
Sa1	Blood culture	0.19 ± 0.01	Low producer	1.42 ± 0.55	High producer
Sa3	Blood culture	0.13 ± 0.02	Low producer	0.76 ± 0.05	Low producer
Sa4	Blood culture	0.59 ± 0.05	Low producer	0.9 ± 0.22	Low producer
Sa16	Blood culture	0.22 ± 0.03	Low producer	0.66 ± 0.04	Low producer
Sa17	Blood culture	0.17 ± 0.03	Low producer	1.39 ± 0.07	High producer
Sa18	Blood culture	2.62 ± 0.39	High producer	2.76 ± 0.15	High producer
Sa21	Blood culture	0.24 ± 0.06	Low producer	1.46 ± 0.2	High producer
Sa26	Blood culture	0.21 ± 0.01	Low producer	1.58 ± 0.2	High producer
Sa5	Superficial pus	0.46 ± 0.05	Low producer	1.01 ± 0.2	High producer
Sa6	Superficial pus	0.26 ± 0.03	Low producer	2.72 ± 0.24	High producer
Sa7	Superficial pus	0.22 ± 0.02	Low producer	1.84 ± 0.13	High producer
Sa13	Superficial pus	0.15 ± 0.02	Low producer	3.1 ± 0.4	High producer
Sa24	Superficial pus	0.62 ± 0.08	Low producer	1.64 ± 0.23	High producer
Sa25	Superficial pus	0.12 ± 0.01	Low producer	0.17 ± 0.05	Low producer
Sa28	Superficial pus	0.14 ± 0.02	Low producer	0.53 ± .0.02	Low producer
Sa29	Superficial pus	0.14 ± 0.03	Low producer	2.46 ± 0.07	High producer
Sa30	Superficial pus	1.00 ± 0.08	High producer	3.25 ± 0.05	High producer
Sa31	Superficial pus	0.18 ± 0.04	Low producer	2.97 ± 0.07	High producer
Sa32	Superficial pus	0.16 ± 0.07	Low producer	3.27 ± 0.07	High producer
Sa9	Deep pus	0.15 ± 0.01	Low producer	1.58 ± 0.05	High producer
Sa27	Deep pus	0.14 ± 0.03	Low producer	1.1 ± 0.04	High producer
Sa2	Various	0.12 ± 0.02	Low producer	0.28 ± 0.03	Low producer
Sa8	Various	0.42 ± 0.13	Low producer	2.58 ± 0.04	High producer
Sa10	Various	0.80 ± 0.30	Low producer	1.91 ± 0.09	High producer
Sa12	Various	2.96 ± 0.11	High producer	3.38 ± 0.12	High producer
Sa15	Various	1.77 ± 0.60	High producer	2.22 ± 0.04	High producer
Sa19	Various	0.15 ± 0.01	Low producer	0.51 ± 0.03	Low producer
Sa23	Various	0.13 ± 0.01	Low producer	0.15 ± 0.02	Low producer

**Table 4:** Effects of different concentrations of *M. alternifolia* essential oil and terpinen-4-ol on *S. aureus* biofilm formation on polystyrene and glass strips.

Strains		Biofilm on Polystyrene		Biofilm on Glass	
		*M. alternifolia*	Terpinen-4-ol	*M. alternifolia*	Terpinen-4-ol
	MIC	7.63 ± 0.73	74.62 ± 1.9	42.35 ± 9.89	28.58 ± 1.78
Sa12	2 × MIC	21.15 ± 1.91	78.03 ± 2.26	54.63 ± 1.51	68.37 ± 1.06
	4 × MIC	26.48 ± 2.52	83.27 ± 2.51	66.56 ± 1.69	75.45 ± 2.43
	MIC	31.67 ± 2.21	83.95 ± 4.44	4.5 ± 2.47	56.43 ± 9.26
Sa15	2 × MIC	44.57 ± 1.81	89.26 ± 3.37	20.45 ± 6.08	72.75 ± 0.92
	4 × MIC	59.05 ± 2.83	91.24 ± 5.81	40.85 ± 1.61	73.79 ± 9.47
	MIC	4.92 ± 6.75	83.35 ± 7.11	23.42 ± 2.19	56.91 ± 2.26
Sa18	2 × MIC	19.59 ± 3.26	87.39 ± 4.37	28.51 ± 1.42	57.35 ± 2.34
	4 × MIC	27.74 ± 1.76	91.03 ± 5.05	49.09 ± 1.55	62.76 ± 1.26

Data are the mean of three replicates ± SD.

**Table 5:** Qualitative violacein inhibition on *C. violaceum*. ATCC 12472.

Concentration	% of Violacein Inhibition	
	*M. alternifolia*	Terpinen-4-ol
**MIC**	69.3 ± 2	34.74 ± 1.6
**MIC/2**	58.98 ± 1.7	18.04 ± 0.1
**MIC/4**	45.74 ± 1.3	17.87 ± 2
**MIC/8**	37.15 ± 1.8	1.47 ± 1
**MIC/16**	31.34 ± 2.3	1.32 ± 1.3
**MIC/32**	23.55 ± 1.7	1.02 ± 0.9

MIC *M. alternifolia* = 10 mg/mL; MIC terpinen-4-ol = 1.25 mg/mL.

**Table 6:** Effect of TTO and terpinen-4-ol at different concentrations (50, 75 and 100 µg/mL) on swarming motility of PAO1.

Component	Concentrations		
	50 µg/mL	75 µg/mL	100 µg/mL
**TTO oil**	16.67 ± 0	25 ± 1.17	33.33 ± 1.09
**Terpinen-4-ol**	25 ± 0	25 ± 0	25 ± 0

## Supplementary Materials

Supplementary File 1
