# Supplementary material for: Chromobacterium violaceum and Pseudomonas aeruginosa PAO1: Models for Evaluating Anti-Quorum Sensing Activity of Melaleuca alternifolia Essential Oil and Its Main Component Terpinen-4-ol
Source: Molecules. 2018 Oct 17;23(10):2672. doi: 10.3390/molecules23102672 (PMC6222492; doi:10.3390/molecules23102672)
Supplement: Supplementary File 1 [file molecules-23-02672-s001.pdf]

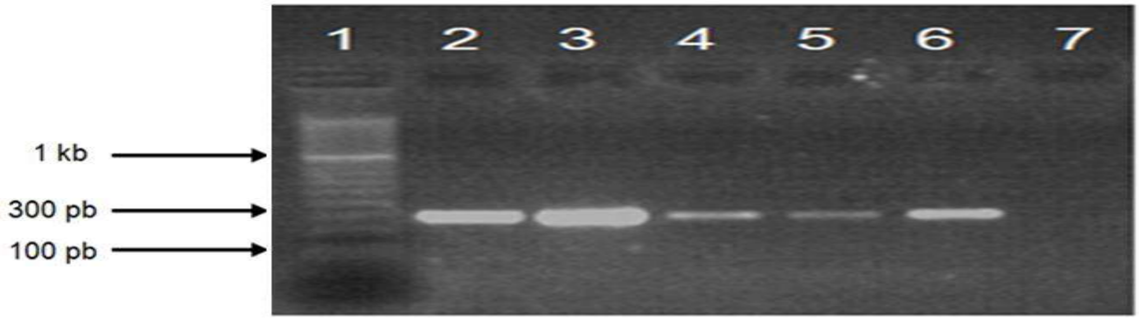

Fig. 1. Agarose gel electrophoresis (1% agarose) of the amplification products obtained for *mecA* gene. (M): molecular weight marker (100-pb DNA ladder, Invitrogen). Lanes 1-2: Positive control *S. aureus* strains (ATCC 6538 and ATCC 43000); lanes 4-6: representative *S. aureus* strains (Sa4, Sa24, Sa15); lane 7: negative control (*S. epidermidis* CECT 231).
